# Medical students in Sudan: an urgent need for support

**DOI:** 10.11604/pamj.2023.46.88.37445

**Published:** 2023-11-21

**Authors:** Haitham Mohamed El Bingawi

**Affiliations:** 1 Department of Medicine, Sultan Qaboos University, Al-Khoud, Oman

**Keywords:** Medical students, Sudan, academic, non-academic, support

## Abstract

Medical students are smart enough to be dismissed for academic reasons. Unfortunately, without support, they do. In Sudan, the statistics are deficient on how medical schools provide support to their students, and as the country marches towards an exciting new future of development, it is important to ensure launching in the right direction. Based on the literature search and the author's experience as a medical student, clinician, and medical educator, the difficulties facing medical students in Sudan were explored; and the required roadmap for support is suggested. Under-resourced facilities, staff migration, lack of robust student support service, and limited access to authentic databases are major problems facing medical students in Sudan. The support roadmap should come at three complementary levels: governmental, institutional, and community. National interventions need to be established to impose standards that universities must observe to ensure that students receive minimal support. The suggestions discussed in this article provide a roadmap for any medical school in Sudan whether it is facing these challenges or simply wants to improve its students' educational environment.

## Essay

Sudan is one of the most geographically diverse states in Africa [1]. It has a special geopolitical location with a multicultural and multi-ethnic society. As a result, diverse populations of students are presented to the doorsteps of universities every year. Entry into medical schools is highly competitive; it depends on students´ performance in the high school exit examination. The Ministry of Higher Education and Scientific Research adopts a central electronic system of application to all universities and higher institutions. There is a strict quota limiting the number of students who get accepted into public medical schools. Students must attain high scores; they are usually the cream of the cohort of students sat for the final secondary exam. However, during their college training, it's not unusual to see these smart students suffer from, depression, somatic symptoms, burnout, broken relationships, and suicidal attempts [2-5]. More despondently, fail their exams and get dismissed because of declining academic performance. The literature identifies several factors specific to studying medicine that might result in reduced academic performance, or lead to psychological, mental, and emotional stresses. These factors are but are not limited to, changes in the social environment, workload, time pressure, information overload, exams, fear of failing, and financial hardship [3,5-7]. Unfortunately, in Sudan, students suffer from additional challenges that cause more stumbling and suffering.

**Objectives:** this paper describes the challenges facing medical students in Sudan and highlights the roadmap for the solution.

### Challenges facing medical students in Sudan?

**Under-resourced facilities:** in 1990, many new medical schools were established because of what is named ´the higher education revolution´. These schools are distributed across the country states. The idea behind their establishment was not only to add on the very few but also to support the community´s health needs. It's now more than thirty years since their establishment. These schools reduced the health disparity through a fairer distribution of the health workforce in their region. Furthermore, many of them have succeeded in facing multiple challenges through innovations in different aspects [8]. However, despite the efforts, they continued to face challenges like reduced physical infrastructure, reduced staffing, and the inevitable medical migration, the paper by Fahal explores these issues in detail [9]. Medical migration sometimes described as “medical brain drains” produces a serious shortage of healthcare professionals in Sudan. According to a national policy response, in 2015, over 15,000 Sudanese physicians were practicing in Saudi Arabia alone and over 3000 are working in the UK and Ireland [10]. Most of the academic immigrants were active employees before their immigration [11]. The remaining staff because of the workload are placed under pressure and are left with few opportunities other than to seek and provide their services elsewhere. Migration, therefore, brings many disadvantages to Sudan, as the migrants leave a huge workforce gap, not only concerning health care practice and service, but also in terms of teaching, mentoring, research, and administrative duties. This view is supported by Gibbs [12].

**Lack of comprehensive and robust student support and wellbeing services:** as defined by Kaur (2016), student support services are the academic and non-academic clusters of services provided by the institute to improve the quality of students´ learning experience. Unfortunately, students support service is the ignored side of higher education in Sudan. At the beginning of the higher education revolution, the National Student Welfare Fund was established to provide support to needy students during their studies. It is a partnership between society and the government. Inappropriately, the Fund´s role has mainly declined in providing accommodation services to needy students ignoring the other academic and non-academic clusters of services required by a wide range of students. It has been said: “*If the institute is deficient in providing support to its students, then it does not serve the purpose of education, but the only distribution of degrees*” [13]. Many junior students initially don´t know what specialization they want to pursue in the future. However, as they develop in their training, certain factors shape their specialty preferences and future career choices like financial rewards, job opportunities, academic interests, competencies, the future quality of life, and advice from others [14,15]. Career advice service is very ignored in most medical schools in Sudan. As a result, the graduates' preferences for future specialties, on many occasions, are not consistent with the population´s health needs or the future demands of the workforce. Stakeholders, need to develop strategies that help align students' performance with the health needs of the country [16].

**Limited access to authentic databases:** added to the low number of teaching staff, limited resources, and lack of professional career advice, students are faced with two problems- limited access to authentic databases and the rapid explosion of medical information. How do medical students survive disease updates, guidelines, drug data, and clinical round information without access to authentic data or advice on how much to take and from where? In their study, Ahmed *et al*. investigated the pattern of use of the Internet by medical students in a private medical college in Sudan, they found that only 39% of the students owned a personal computer at home, and approximately 15% were connected to an internet server [17]. The rest access the internet from an internet café! They found that most of their students rated themselves as having poor skills in navigating the internet. To me, this is the situation of most of our medical students. Even if they get access to the internet, they lack the competence to judge the credibility of the data they get, and it is illogical to view this in reverse. Students face a hard time distinguishing credible from non-credible information or identifying where the information came from [18]. On the other hand, students face the global problem of information overload. Beker *et al*. point out that when students are faced with a lot of facts to remember, they are left with two options: to learn everything or to learn only the most important [19]. The question is what is the most important? Unfortunately, this is often left to the student´s judgment. In a study done by McMillan, a student was quoted as: “…*I mean, for them to tell you ´go read up´, that for me is just throwing us further away*" [20]. Such a comment indicates that students are struggling to know to what depth a particular topic is to be understood. There should, therefore, be a way to highlight the information around which students can structure their learning, and more importantly, provide them with access to the appropriate sources. Furthermore, train them to handle the available information without experiencing an unpleasant sense of disorientation.

**Figure 1:**
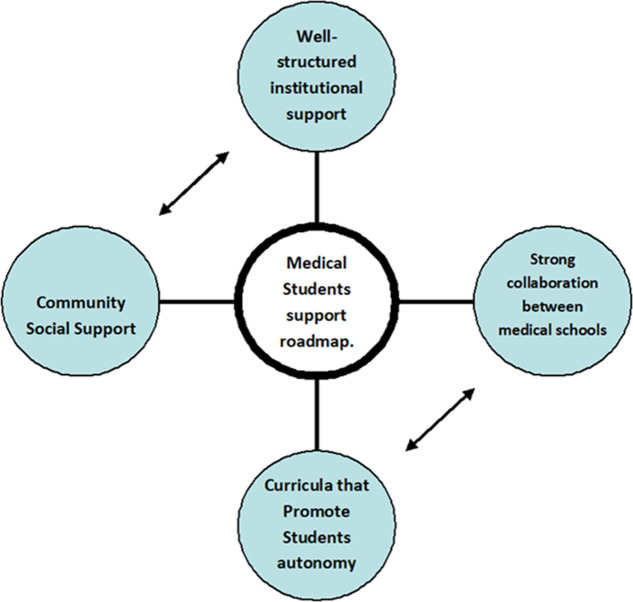
medical students support roadmap in Sudan

**The support roadmap:** many students find the way on their own without much support in this highly demanding lifelong medical career. However, as a matter of concern, many of them have failed to complete the medical degree within the required timeframe. It's perhaps the time to seriously reflect on the level of support provided to our students. There is no easy way out, particularly, with their very diverse needs in a country facing many challenges. It may be logical to present the roadmap for support at different levels, governmental, institutional, and community. Here are some suggestions that might help decision-makers make a forward step toward helping and supporting medical students in Sudan, shown dramatically in Figure 1.

### Governmental and institutional roles

**Establishment of comprehensive, and robust student support and well-being services:** to improve the educational achievements of our students, there should be a support program tailored to their diverse needs. The services provided should include, but are not limited to, quality academic counseling, learning assistance service, mentoring service, career guidance, personal development advice, and financial and health counseling services. For example, career counseling services can help students distinguish the different features of all specialties in depth so they can make their final decisions more wisely [14]. Such counseling services should be available very early, perhaps from day one in medical school. The staff selected to work in the support service program should have good training to perform the required tasks. Students´ readiness to discuss personal/academic problems with their tutors depends upon the formation of a good relationship and it is this relationship that is most related to the perceived success of the scheme [21]. Therefore, staff development should be one of the major responsibilities of the program. The system should ensure that all tutors and staff agree on the support framework otherwise it will not be flexibly delivered. As the aim of this system is to ensure appropriate referral, provide access to the appropriate support service, and secure the continuation of the program, monitoring is important. This can be done through regular students´ and staff monitoring questionnaires. The office should prepare student and staff guidance booklets for both students and staff and sure that the service meets the core university academic standards. For it to succeed, the support office should work closely with other university departments. This may ensure support from both the senior persons in the universities and the Ministry of Higher Education.

**Furthering the roles of the student union:** student union in universities has a prominent role in achieving the vision and mission of universities, as it is the link between students and the administration. The union carries out a wide range of activities and events that differ according to the objectives of the union and the interests of its students. These roles can expand further to effectively help in transforming the educational experience towards more support, fun, effectiveness, and innovation. For example, the union can play a crucial role in blending the academic demands of students by asking distinguished graduates in certain disciplines to provide free-of-charge classes. It can operate as a non-profit charitable organization that collects donations or negligible fees from members to be used to solve financial problems. Not only do student unions help charitably solve academic or financial constraints, but they also can establish partnerships with institutions specialized in supporting students.

**Establishing an official network association between medical schools:** in our context, creating a network association between different medical schools, preferably between the established and the new one, to collaborate, work together, and share resources will play an important role in enriching students' educational experience. New medical schools will benefit from the many resources of well-established ones. Additionally, schools can learn from each other the key issues specific to health education in the wide areas of Sudan. The experience of a partnership between universities was a success in many African medical schools [22,23]. For example, the Consortium of New Southern Africa Medical Schools (CONSAMS) sought to support each other through, resources, sharing of faculty, and innovative programs [23]. In Sudan, efforts should be made to adopt a model of a consortium that guarantees benefits for all parties. For example, establishing a research group under clear rules and regulations, will upgrade the technical know-how of doing research and facilitate learning from expert researchers. Another suggestion is to establish a student exchange program that provides students with the opportunity to learn from the school with whom to exchange. Additionally, the institutes may establish a central digital library that can be accessed by the computer network. However, for this to succeed and be effective, medical schools should secure a good number of computers and excellent internet facilities. There are several models for collaboration, only limited by the imagination of the leaders. In addition to its role in promoting the high standards of medical education in Sudan and in supervising the exams that qualify for medical degrees from the various Sudanese universities, the Sudan Medical Council can operate as the coordinating body between the universities in the aspects suggested earlier.

**Promoting students' autonomy:** more than 100 years ago, William Osler (1849-1919) said: “*…to cover the vast field of medicine in four years is an impossible task*”. He also continued to say: “*The hardest conviction to get into the mind of a beginner is that the education upon which he is engaged is not… a medical course, but a life course, for which the work of a few years under teachers is but a preparation… whether you will falter and fail in the race or whether you will be faithful to the end depend on the training from the start and on your staying power*” [24]. This addresses the fact that learning does not end with basic training in medical schools, but simply, continues for life [25]. It also addresses as well, the importance of helping students acquire the competencies that help them to grow and develop throughout their careers. In self-directed learning, students make use of the available resources and take good control over their learning. It's a key issue in adult learning' [26]. Perhaps, with appropriate direction, tutor guidance, and institutional support it is an ideal model in our context. On the other hand, Glasziou *et al*. are quoted as saying: “*a 21^st^-century clinician who cannot critically read a study is as unprepared as one who cannot take blood pressure or examine the cardiovascular system*” [27].

Hence, learning how to practice evidence-based medicine (EBM) is a crucial skill for self-directed learning. Medical students should be provided access to a free-of-charge source of 'authentic science'. They should be trained in Evidence-based medicine (EBM). More precisely, to formulate answerable clinical questions and approach databases using search engines. They should acquire the skills needed for the evaluation and critical- appraisal of scientific information. The importance of students fishing by themselves, rather than being spoon-fed by instructors is a crucial competence that enables students to be autonomous in distinguishing the best available evidence from within a large body of research. Lastly, our curricula should be redesigned to foster the development of self-directed learning skills by incorporating EBM in teaching and assessment like what we do in our daily clinical practice [27]. Furthermore, curricula should step up to give more space and value to elective courses and research.

**Community engagement:** as medical schools have a responsibility to promote health in the local communities, the communities should play an active role in supporting and augmenting medical education. It's argued that students' levels of engagement in the community were dependent upon communities promoting a sense of belonging and providing positive learning experiences [28]. Therefore, local communities should provide the necessary social support when students are in community training. Social support has a great role in relieving those who fall under the stress of studying medicine and is considered one of the important sources of security for the student´s needs in their academic achievements. The support can take different forms, for example, through non-governmental community organizations that contain qualified social workers who through knowing the nature of societies can provide the necessary support to students. Or through involving community leaders in the university governing structure.

## Conclusion

In Sudan studying medicine requires more than exceptional academic performance. There appears to be a lack of structured institutional support. Without these students will continue to suffer. National interventions need to be established to impose standards that universities must observe to ensure that students receive minimal support. The suggestions discussed in this article provide a roadmap for any medical school in Sudan whether it is facing these challenges or simply wants to improve its students' educational environment.

**Practice points:** i) under-resourced facilities, staff migration, lack of robust student support service, and limited access to authentic databases are major problems facing medical students in Sudan; ii) the support roadmap should come at 4 complementary levels: governmental, institutional, and community. It should be tailored to the diverse nature of our medical students with to-the-purpose trained staff; iii) establishing a network association between medical schools to collaborate, work together, and share resources will play an important role in enriching students' educational experiences; iv) curricula should be redesigned to foster the development of self-direction; v) the local communities should play more positive roles in the social support of medical students.
